# Differential effects of coral-giant clam assemblages on biofouling formation

**DOI:** 10.1038/s41598-019-39268-1

**Published:** 2019-02-25

**Authors:** Isis Guibert, Isabelle Bonnard, Xavier Pochon, Mayalen Zubia, Christine Sidobre, Gaël Lecellier, Véronique Berteaux-Lecellier

**Affiliations:** 1 Sorbonne Université, Collège Doctoral, F-75005 paris, France; 2 USR3278 PSL CRIOBE CNRS-EPHE-UPVD, LabEx CORAIL, Papetoai, Moorea French Polynesia; 3 UMR250/9220 ENTROPIE IRD-CNRS-UR, LabEx CORAIL, Promenade Roger-Laroque, Noumea cedex, New Caledonia France; 4 USR3278 PSL CRIOBE CNRS-EPHE-UPVD, LabEx CORAIL, Université de Perpignan, 58 avenue Paul Alduy, 66860 Perpignan, France; 5 Coastal and Freshwater Group, Cawthron Institute, Private Bag 2, Nelson, 7042 New Zealand; 6 Institute of Marine Science, University of Auckland, Private Bag 349, Warkworth, 0941 New Zealand; 7 University of French Polynesia, UMR-241 Ecosystèmes Insulaires Océaniens, BP 6570, 98702 Faa’a, Tahiti French Polynesia; 8 Université de Paris-Saclay, UVSQ, 45 avenue des Etats-Unis, Versailles Cedex, France

## Abstract

To prevent the settlement and/or the growth of fouling organisms (i.e. bacteria, fungi or microalgae), benthic sessile species have developed various defense mechanisms among which the production of chemical molecules. While studies have mostly focused on the release of chemical compounds by single species, there exist limited data on multi-species assemblages. We used an integrative approach to explore the potential interactive effects of distinct assemblages of two corals species and one giant clam species on biofouling appearance and composition. Remarkably, we found distinct biofouling communities suggesting the importance of benthic sessile assemblages in biofouling control. Moreover, the assemblage of 3 species led to an inhibition of biofouling, likely through a complex of secondary metabolites. Our results highlight that through their different effect on their near environment, species assemblages might be of upmost importance for their survival and therefore, should now be taken into account for sustainable management of coral reefs.

## Introduction

Benthic sessile communities are under direct and constant pressure of their surrounding environment. Therefore, many species have developed various mechanisms such as direct and indirect biotic interactions to maximize their survival^1^. Each of these interactions can be either positive or negative and are responsible to a large extent for structuring communities^2^. Most studies on bioactive products have focused on the competition and defense processes that may lead to the discovery and commercialization of new bioactive metabolites^3^.

In sessile coral reef organisms, numerous studies have identified a wide variety of secondary metabolites such as terpenoids (e.g. siphonodictine^4^), saponins (e.g. muricins^5^), macrolides (e.g. latrunculins^6^), and steroids (e.g. verumbsteroids^7^) that can reduce the growth or even lead to the death of neighboring species. The production of such secondary metabolites, mainly non-polar, synthesized constitutively or inducible^8^, strongly influences sessile organisms’ behaviors in response to local competition or predation pressures. The latter are particularly pervasive in marine biofouling environments where strong developmental and organism-environment interactions take place^9^.

Biofouling is an ubiquitous phenomenon in the marine environment and a common feature of a wide variety of natural and artificial structures^10^. Biofouling formation typically starts with the adhesion of dissolved organic matter onto a surface which leads to physiochemical changes^11^ and to the development of a bacterial mat or early biofilm layer^12^. Successfully settled bacterial communities may influence the successive settlement of micro-algae (e.g. diatoms, cyanobacteria), fungi and protists, which are all precursors for colonization by larger fouling organisms or macrofouling^9^.

Biofouling formation onto a substrate, its growth rate and the type of species able to colonize this habitat are modulated by various interactions and involve complex biochemical, behavioral or physical mechanisms^9,13^. The external environment plays an important role in the biofouling formation, in particular through physical and chemical conditions^14,15^. For example, the availability of nutrients will play a critical role in the selection of early colonizers and the successive community composition of the biofouling^16^. Light and temperature increase also play important roles in enhancing the propagation of biofilm and biofouling^17,18^. Besides these environmental factors, many antifouling agents have been characterized from sessile marine organisms, in particular antifouling molecules produced by sponges, soft corals, and seaweeds^12,19,20^. Microorganisms associated with marine algae and invertebrates, such as epibionts, also possess antifouling potential^12^. Despite the growing knowledge that biofouling-associated antifouling compounds are damaging to both larval and adult stages of hard corals and giant clams^21–23^, there exist very limited data on the actual processes and the specific molecules involved in antifouling activities of multiple interactive coral reef species. There is now growing evidence that hard corals release antifouling active substances^3^, which can confer different competitive abilities to coral species against algae^24^.

Studies on Antarctic marine benthos have shown that by bringing together different defense strategies, mainly regulated by chemical interactions such as deterrent or repellent molecules, species assemblages create a complex model of interaction which may help protect Antarctic organisms from competition, for space and fouling pressure notably^25^. In coral reef ecosystems, the variety of antifouling compounds produced by sessile organisms, even among coral genera, prompted us to study the importance of species biodiversity and interaction on coral reefs’ fitness. Our work investigated their potential cumulative effect on biofouling formation and their ability to minimize the dramatic impact that uncontrolled biofouling expansions (i.e shifts from coral to algal dominated reefs) may have on coral reef ecosystems.

Thus, in this study we combined cytological, metagenomic and metabolomic approaches to explore the interactive effects of multiple benthic species assemblages on the biofouling appearance and composition under normal and thermal stress conditions. Two common Indo-Pacific scleractinian coral species, *Pocillopora damicornis* and *Acropora cytherea*, and the giant clam *Tridacna maxima* were artificially grouped in distinct assemblages, exposed to varying water temperatures, and their influence on algal biofouling formation was investigated.

## Material and Methods

### Sample collection and preparation

#### Corals and giant clams collection

*Acropora cytherea* (n = 4) and *Pocillopora damicornis* (n = 4) colonies were collected at low tide (∼2 m) in Moorea lagoon, French Polynesia (17°30′S, 149°50′W fringing reef Linareva^26^). The sampled colonies were then cut into nubbins using side-cutting pliers to produce a minimum of 45 small fragments each. All the samples were grown on tables in a common garden located in the lagoon area of the InterContinental Moorea Resort & Spa during an 8 months period. The giant clams *T. maxima* were purchased at a French Polynesian distributor on Reao Island (18°28′S, 136°25′W; Company identification number - N°Tahiti: 139 519), and then acclimated in the common garden. The three species were placed on 3 different distant (>3 m) tables in order to avoid inter-species’ interactions. A CITES permit was obtained to allow for samples export (CITES – FR1698700087 – E).

#### Experimental design

One to three species were placed in aquariums. The different associations of organisms per aquarium are thereafter referred to as “assemblage”. Five distinct assemblages of coral and/or giant clams species were studied: *P. damicornis* + *A. cytherea* + *T. maxima* (PAT); *P. damicornis* + *A. cytherea* (PA); *A. cytherea* + *T. maxima* (AT); *P. damicornis* (P) or *T. maxima* (T) alone. Three nubbins from 4 coral colonies per species (12 nubbins per coral species) and/or 12 giant clams were used for each assemblage. Therefore, the 40 L aquariums were composed of either 12 (assemblages T and P) or 24 (assemblages PA and AT) or 36 (assemblages PAT) organisms (Supplementary S1a). For each experiment (A, B and C), one aquarium without macroorganisms was kept as a control at lagoon temperature (W). Aquariums were randomly chosen to avoid a potential aquarium effect. Each aquarium was open-circuit with seawater pumped directly from Moorea Opunohu’s bay. Two filters of 60 microns each were used to remove sediment, and the water flow in aquariums was 20 L/h. The water temperature within aquariums was controlled with the Biotherm pro system (Hobby, Stukenbrock, Germany). Temperature/Light Data Loggers (P/N U22-001, Onset, Bourne, Massachusetts; or Ruskin, Ottawa, Canada) recorded temperature data every 10 minutes. All aquariums received the same light using LED lamps (PlanetPro ELOS, Verona, Italy), following a diurnal cycle.

The experiment was performed in duplicate (exp. A and B), spaced two weeks apart, except for the AT assemblage which was also studied in duplicate but during the same experiment (exp. C). In each of A and B experiments, 4 distinct assemblages were studied in parallel (assemblages PAT, PA, T and P; 2 aquariums per assemblage; n = 8 aquariums per experiment). After an acclimation period of 12 days, for each set of assemblage, one of the two aquariums was maintained at the ambient lagoon temperature (27 °C; LT; control), and the second one was submitted to a stress temperature (ST). The stress temperature was performed as follows: increased 1 °C per day from day 12 to day 17 up to 32 °C where it was maintained for 3 days (from day 17 to day 19), before being decreased back to 27 °C (day 20). Similarly, in experiment C, after the acclimation period, two aquariums of AT assemblages were placed at lagoon temperature and the remaining two were exposed to the stress temperature (n = 4 aquariums).

#### Biofilm and nutrient sampling

During the experiments, biofilm deposited on each aquarium wall was scraped at day 12 and 20 using a cover glass slide, and stored in 70% ethanol at −20 °C until further DNA analysis. Three replicates of water samples (40 mL) from aquariums were sampled at the same time and kept at −20 °C until the analysis of nitrite (NO_2_^−^), nitrate (NO_3_^−^), phosphate (PO_4_^2−^), ammonium (NH_4_^+^) and silicate (Si-OH_4_; Technicon Autoanalyzer system, Southampton, United Kingdom). We chose to analyze samples from day 12 (T0) during the experiments A, B and C; samples from day 17 (T1) during experiment B and from day 19 (T2) samples during experiment B and C. A synthesis of the tested samples as well as the statistics used are on Supplementary S1b and S1c.

#### Biofouling observation and cytology

Throughout the experiments, photographs of the aquarium were taken (days 15 or 19) and daily observations of the biofouling were recorded.

At beginning of each experimental run, two sterile microscope cover glasses and two sterile glass slides were placed in each aquarium. At the end of the experiment, the slides were observed in order to identify the most predominant genera per assemblage (Supplementary S1c). For the aquarium without macroorganisms (W), two slides for experiment B and C were observed.

#### DNA extraction, PCR amplification and sequencing

Biofilm DNAs were extracted using the CTAB-based protocol described in Rouzé *et al*.^27^ and stored at −20 °C until PCR amplification. Two different genetic markers were selected for metabarcoding analysis. The 16S ribosomal RNA V3/V4 region was amplified with the Bakt_341 forward (5′-CCTACGGGNGGCWGCAG-3′) and Bakt_805 reverse (5′-GACTACHVGGGTATCTAATCC- 3′) primers^28^. The 18S ribosomal RNA V4 region was amplified with the primer pair D512for18S forward (5′-ATTCCAGCTCCAATAGCG-3′) and the D978rev18S reverse (5′-GACTACGATGGTATCTAATC-3′), specifically designed for characterizing diatom communities^29^. The primers were modified to include Illumina^TM^ overhang adaptors as described by Kozich *et al*.^30^.

The polymerase chain reactions were performed (Supplementary S1c) and products were visualised on a 2% agarose gel. Samples were purified using magnetic beads (Agencourt Bioscience Corporation, Grand Rapids, Michigan), quantified (Qubit® 2.0 Fluorometer, Invitrogen, Carlsbard, California) and diluted at equimolar concentration to 3 ng/µL. Amplicons from DNA samples previously obtained from two high-latitudinal invasive species (*Ciona savignyi* and *Asterias amurensis*) from a previous study^31^, were used as internal quality controls. Nuclease-free UltraPure™ water (Thermo Fisher Scientific, Waltham, Massachussetts) was used as negative control. The samples were sent to New Zealand Genomics Limited at the University of Auckland for final high-throughput sequencing library preparation. Libraries were sequenced on a MiSeq Illumina™ platform using a 2 × 250 base-pair (bp) paired-end protocol.

#### Metabolomic sample preparation and Liquid Chromatography-Mass Spectrometry (LC-MS) analysis

Seawater samples from input pipes (n = 5) were collected through a tap placed before the aquariums, and seawater samples from aquariums (n = 23) were individually siphoned and filtered using cartridges Strata-XL 100 u polymeric reversed phase (Phenomenex, Torrance, California). Six liters of seawater per sample were filtered using an extraction chamber.

Samples were extracted from the Strata-XL cartridges and then centrifuged to separate the residual water. The organic phase was transferred in a glass hemolysis tube before drying under nitrogen flow and freeze-dried overnight. Before LC-MS analysis, organic extracts were prepared at a concentration of 1 mg/mL in methanol and filtrated on Uptidisk (PTFE, 0.2 mm, Interchim, San Diego, California).

LC-MS analyses were performed with an LCQ Fleet 2300 (Thermo Scientific) to determine metabolites in the seawater of each assemblages. The samples were analyzed using a positive ion mode with full scan MS window of 200-2000 *m/z*. A quality control strategy was carried out using a Latin square design for samples, quality control and methanol blank samples in order to reduce the potential error of instrumental drift.

To determine the exact mass of secondary metabolites selected, LC-HRMS analyses were performed on a UHPLC system (Vanquish, Thermo Scientific) interfaced to a QTOF mass spectrometer (MaxisImpact I, Bruker Daltonics, Billerica, Massachussetts). Finally, to strengthen the putative identification of selected metabolites, LC-HRMS and LC-HRMSMS analyses were performed on a UHPLC system (Vanquish, Thermo Scientific) interfaced to an Orbitrap mass spectrometer (Q-exactive Plus Hybrid, Thermo Scientific, Supplementary S1c). Finally, metabolites were assigned according to literature and databases such as LIPID MAPS, Metlin or MarinLit^32–34^.

Cytology, nutrients, metabarcoding and metabolomics methods are detailed in the Supplementary File S1c.

### Experiments

All experiments were performed in accordance with relevant guidelines and regulations.

## Results

### Significant change of seawater composition between experiments but only slightly between assemblages

The principal component analysis of the seawater nutrients (Supplementary Table S2 and Figure S3) enabled to distinguish each experiment. Nutrients were more abundant in the experiments A and C than in the experiment B, and Si-OH_4_ were more abundant in the experiment A compared to the two others. For each experiment, slight significant variations of nutrient concentrations were observed between conditions (stress-lagoon temperature) or between assemblages. Whatever the experiments, NH_4_^+^ and NO_2_^−^ concentrations were systematically higher in all assemblages containing giant clams (Tukey test p < 0.05). Moreover, NH_4_^+^ concentrations were higher in PAT assemblages than in T assemblages. However, while in the experiment A, the range of NH_4_^+^ concentrations in PAT and T assemblages were of [1.493-3.016] µmol/L and of [1.059-1.287] µmol/L, respectively, NH_4_^+^ concentrations in the experiment B were less than 1 µmol/L (except for 1 sample) in PAT assemblages, which is lower than the range in T assemblage of the experiment A. Overall, the differences of water composition (relative and absolute concentrations) within experiments remained lower than the differences of water composition between experiments.

### Difference of Biofouling appearance according to the type of assemblages

**Figure 1:**
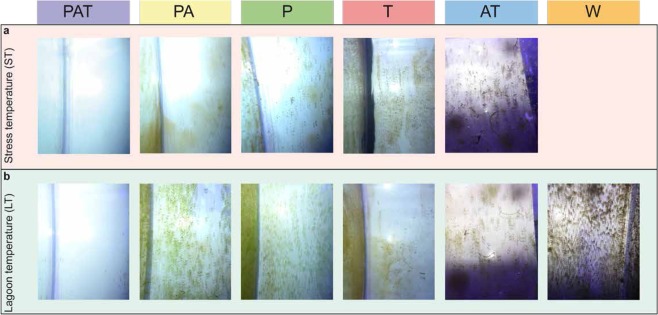
Photographs of aquarium’s walls according to the assemblages (**a**) with or (**b**) without thermal stress. PAT: *P. damicornis*, *A. cytherea* and *T. maxima*; PA: *P. damicornis* and *A. cytherea*; P: *P. damicornis*; T: *T. maxima*; AT: *A. cytherea* and *T. maxima*; W: no macroorganisms; stress temperature (ST); lagoon temperature (LT, control). Photographs: Isis Guibert.

Ten days following the beginning of the acclimation period, the aquarium walls harbored various biofouling aspects that were specifically linked to the type of assemblages under investigation (Fig. 1). This phenomenon was similar between the thermal stress and control conditions (compare ST – LT, Fig. 1), was consistently observed at the end of all experiments and was reproducible. The walls of the aquariums containing PAT assemblage were clean with just a barely perceptible light green biofilm layer. Aquariums containing the T assemblage were covered with a brown biofouling and mucus. Brown and green biofouling were also perceptible on the aquarium walls of the P assemblage, contrasting with only a green layer apparent in the aquariums with either PA or AT assemblages. The most severe biofouling, e.g. murky aquarium walls, appeared in the W aquariums, devoid of macroorganisms. Since no visual difference of biofouling appearance was observed between the two conditions (ST and LT), we chose to focus on the core community by assemblage in order to characterize the different phenotypes observed. Therefore, all data were pooled by assemblage without consideration of treatments.

### Distinct taxonomic groups according to the assemblages

**Figure 2:**
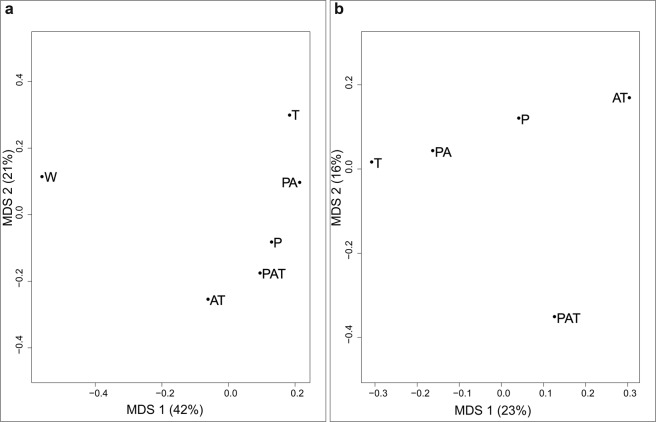
Classical multidimensional scaling from the Jaccard’s index of assemblages with (**a**) and without (**b**) control. PAT: *P. damicornis*, *A. cytherea* and *T. maxima*; PA: *P. damicornis* and *A. cytherea*; AT: *A. cytherea* and *T. maxima*; P: *P. damicornis*; T: *T. maxima* and W: no macroorganisms (control).

Seven taxonomic groups were defined using cytological observations: Chlorophyta, diatoms (Bacillariophyta), cyanobacteria (Cyanophyta), dinoflagellates (Dinoflagellata), Ochrophyta, Rhodophyta and undetermined. Within these, identifications were assigned at the lowest possible taxonomic level (family or genus). We characterized 201 distinct associated taxa across all assemblages. Classical multidimensional scaling analysis of the 6 assemblages based on the Jaccard’s Index (Fig. 2a) showed a strong dissimilarity (separation on the first axis) between aquariums without (W) and with (PAT, PA, AT, P and T) macroorganisms. The second axis separated the remaining five assemblages from T to AT. After exclusion of the control in the analysis, a similar distribution than the second axis of the first classical multidimensional scaling (from T to AT) was directly observed on the first axis and the second axis mainly separated PAT from all others (Fig. 2b).

### Different family and genus composition of the three main taxonomic groups according to the assemblages

We analysed the biofouling of PAT, PA, P and T assemblages using metabarcoding. After data processing of 1 202 179 paired-end reads, 711 313 contigs (16 505–83 708 per assemblage) with an average length of 420 bp (18S analysis) and 340 076 contigs (3 862–38 158 per assemblage) with an average length of 428 bp (16S analysis) were selected for taxonomic and functional analysis.

As the three main steps of biofouling are successively the development of bacteria, diatoms and algae, we focused on these three groups. The bacterial group composed of 13 847 unique contigs was divided into 44 phyla and 856 genera (Supplementary Table S4). Of the 8 330 unique contigs identified from the 18 S analysis, 6 369 were assigned to the diatoms group (Ochrophyta phylum), which divided into 63 genera (Supplementary Table S5). For the algae analysis, 19 990 unique contigs were assigned into 4 phyla (Chlorophyta, Euglenida, Eukaryota, Phaeophyceae) according to Silva database. From those phyla, 29 genera were identified (Supplementary Table S6).

In order to characterize the biofouling for each assemblage, we first compared the presence of families and genera for the three groups. Amongst the 28 characterized families of bacteria (Supplementary Figure S7), 13 families were found in all assemblages and 3 were found exclusively in the T assemblage (*Methylocystaceae*, *Legionellaceae* and *Hyphomicrobiaceae*). We also noticed that 12 bacterial families were present in 2 or 3 assemblages sharing or not a common species. For example, 3 families (*Oceanopirillaceae*, *Halomonadaceae* and *Granulosinoccaceae*) were found in all assemblages containing *P. damicornis* (PAT, PA and P). Other families were found in 2 assemblages such as *Pseudoalteromonadaceae* in PA and T assemblages but not in the PAT assemblage. Amongst the 17 identified families of diatoms, 12 were common to all assemblages, 2 (undetermined *Ochromonadales* and undetermined *Coscinodiscophytina*) were common to PA and P assemblages and 3 (*Rhizosolenids*, *Dictyochophyceae* and undetermined *Pedinellales*) were found exclusively in the PA assemblage. For the algae group, 28 families were identified and 15 were common to all assemblages. No specific families were determined for the PAT assemblage but 2 were specific to PA (*Fucaceae* and undetermined *Eutroptiales*) and to T (*Ulvophyceae* and *Ceramiaceae*) assemblages. Furthermore, *Peridiniaceae*, undetermined *Rotaliida* and *Florideophyceae* families were identified in all assemblages except for the PAT assemblage (Supplementary Table S4-S6).

**Figure 3:**
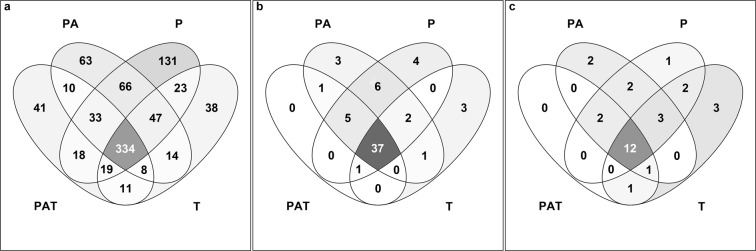
Venn diagrams of genera detected in the assemblages. (**a**) bacteria, (**b**) diatoms (**c**) algae. PAT: *P. damicornis*, *A. cytherea* and *T. maxima*; PA: *P. damicornis* and *A. cytherea*; P: *P. damicornis* and T: *T. maxima*.

At genus level, a qualitative analysis revealed which genera of bacteria, diatom, and algae were common or specific to different assemblages (Fig. 3). Out of the 856 bacterial genera assigned, 474, 576, 672 and 494 were observed in PAT, PA, P and T assemblages, respectively, among which 334 were common. However, 41, 63, 131 and 38 genera were specific to PAT, PA, P and T assemblages, respectively. Interestingly, 47 genera were common to all assemblages except PAT (Fig. 3a). Few diatoms genera were found (63), but similarly to the bacterial group, 37 genera common to all assemblages were found (Fig. 3b). No specific diatoms were observed in the PAT assemblage, but 3 genera in PA, 4 in P, and 3 in T assemblages were specifically observed. Noteworthy, 2 diatom genera (*Melosira* sp. and *Psammoneis* sp.) were common to all assemblages except PAT. Concerning the algae group, only 29 genera were assigned and 12 were common to all assemblages (Fig. 3c). Similar to diatoms, no algal genera were specific to the PAT assemblage, and 3 (undetermined *Peridiniceae*, undetermined *Florideophyceae* and undetermined *Rotaliida*) were common to all the assemblages except this one.

### Characterization of biofouling from each assemblage by specific species abundance and metabolic function of bacteria

**Figure 4:**
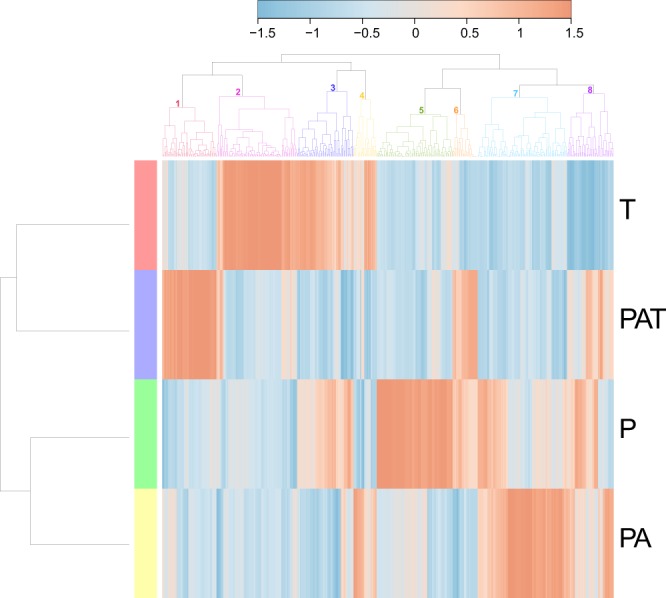
Heatmap based on bacterial genera and showing specific ‘barcode community fingerprints’ for each assemblage. Communities’ differences are showed on a relative scale, with enrichment (red) and depletion (blue) of sequence abundance calculated using the Ward’s clustering method and a Pearson distance measure. T: *T. maxima*; PAT: *P. damicornis*, *A. cytherea* and *T. maxima*; P: *P. damicornis*; PA: *P. damicornis* and *A. cytherea*.

To complement the qualitative analysis, we analyzed the relative abundance of bacteria. All assemblages were dominated by *Rhodobacteraceae* (~25-45%). *Flavobacteriaceae* were also found in large quantities (~5-15%) in all assemblages (Supplementary Figure S7). Nevertheless, the bacterial community composition yielded a specific “barcode community fingerprint” for each assemblage (Fig. 4). As shown, the barcode fingerprint can be clustered into 8 sub-communities. These clusters were rarely found in the same abundance level between assemblages. In fact, high abundances (red color, Fig. 4) of clusters 1, 2 and 5 are specific to PAT, T and P assemblages, respectively.

**Figure 5:**
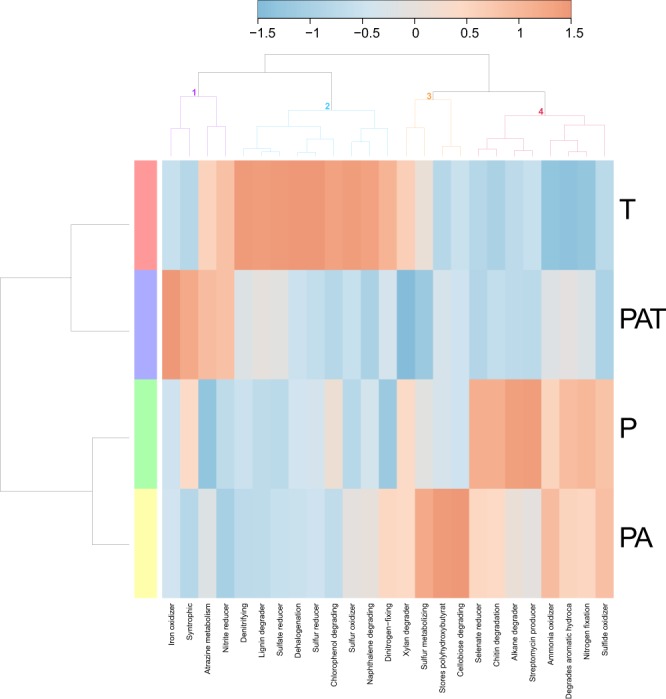
Heatmap of bacterial “metabolism by phenotype”. Data show functional differences derived from each assemblage on a relative scale with enrichment (red) and depletion (blue) of gene functions using a Pearson distance measure and the Ward’s clustering method. T: *T. maxima*; PAT: *P. damicornis*, *A. cytherea* and *T. maxima*; P: *P. damicornis*; PA: *P. damicornis* and *A. cytherea*.

We further compared the metabolic functions of bacterial communities within each assemblage (Fig. 5). Similar to the qualitative analysis, bacterial functions were clustered according to the differential abundance of bacterial species and their putative associated functions. Amongst the 5 clusters, 3 were linked to specific assemblages. Cluster 1 was specific to PAT with an increase of bacteria with “iron oxidizer” and “syntrophic” functions. Cluster 3, where 9 bacterial functions increased, was specific to T, and cluster 4 where “sulfur metabolizing”, “stores polyhydroxybutyrat” and “cellobiose degrading” functions increased was specific to PA. Interestingly, the two other clusters shared similar increase in bacterial functions between two assemblages. Cluster 2 showed an increase in “atrazine metabolism” and “nitrite reducer” functional responses in PAT and T assemblages. Increase of 8 bacterial functions in cluster 5 was common to P and PA assemblages amongst which “selenite reducer”, “chitin degradation”, “alkane degrader” and “streptomycin producer” were strongly represented. Consequently, PAT assemblage was closer to T assemblage than to PA and P assemblages.

### Secondary metabolites released in seawater are related to each assemblage

**Figure 6:**
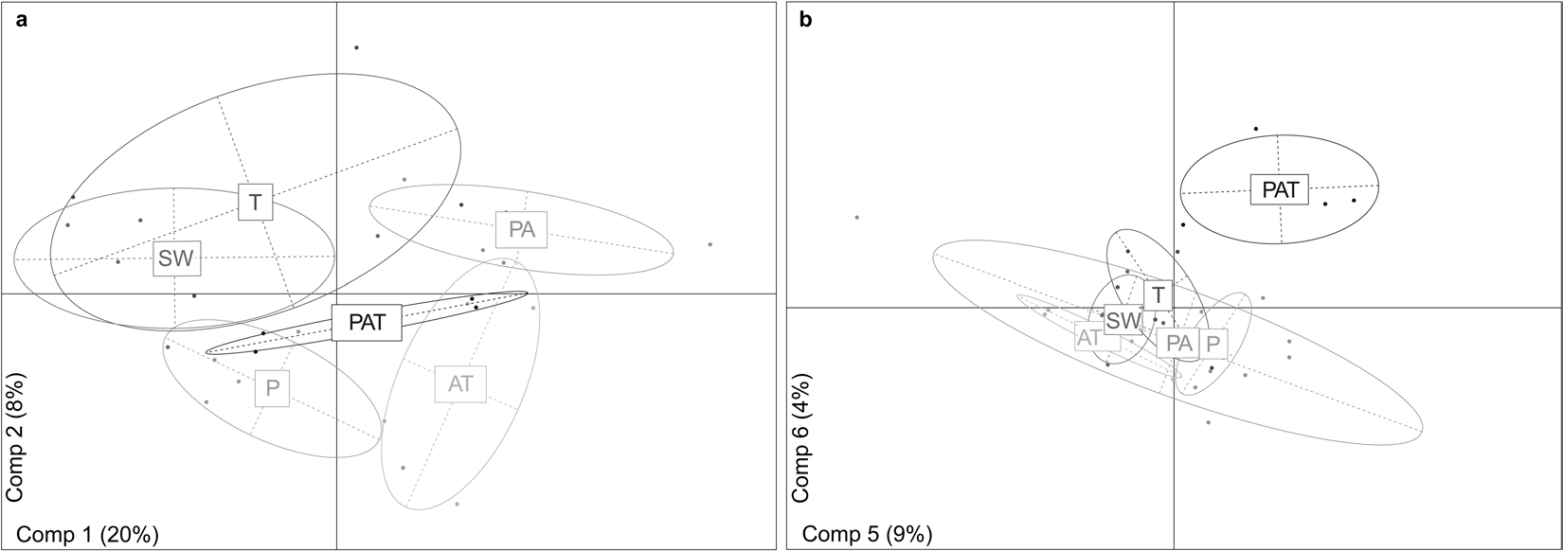
Partial least Square Discriminant Analysis of organic extracts metabolites from seawater’s aquariums. (**a**) Distribution of the metabolites on the first two components (Comp1 and Comp2) allowing the separation according to the assemblages. (**b**) Distribution on the fifth and six components (Comp5 and Comp6) providing the separation of PAT from other assemblages. PAT: *P. damicornis*, *A. cytherea* and *T. maxima*; PA: *P. damicornis* and *A. cytherea*; P: *P. damicornis*; T: *T. maxima*; AT: *A. cytherea* and *T. maxima* and SW: incoming seawater.

To determine if secondary metabolites might account for the difference of biofouling communities, a chemical analysis of the tanks’ seawater was performed. The overall LC-MS analysis allowed for the detection of 1 488 distinct metabolites. A large majority of them (1 370), were detected in both assemblages and incoming seawater. Particularly, no specific metabolites were detected in PAT. A quantitative Partial least Square Discriminant Analysis on metabolites was then conducted to discriminate each assemblage. The first components discriminated every assemblage (Fig. 6a), with PAT at the center of the plot, indicating an average mix of all metabolites rather than the emergence of new metabolites. In order to determine the Variable Importance in the Projection (VIP) potentially contributing to the inhibition of biofouling effect, we focused on the components which are clearly responsible for the separation of PAT from other assemblages (5^th^ and 6^th^ components, Fig. 6b). Thereby, 55 metabolites were selected among which the most abundant were kept as VIPs (n = 28). A similar approach was performed for the less abundant metabolites (68). Nevertheless, none of them were selected because none were specifically less abundant in PAT. The exact mass of VIPs was determined using LC-HRMS analyses with a High Resolutive QTOF mass spectrometer (Supplementary Figure S8). From these masses, the molecular formulas were estimated and putative identifications were assessed for 5 VIPs on 28 most abundant VIPs (Table 1). The 5 VIPs were all detected in each PAT assemblage regardless of the treatment (PAT.L and PAT.S). Identifications were strengthened by full and fragmentation spectra issued from MS analyses with a Qexactive mass spectrometer. According to available literature and databases, VIPs were assigned to carotenoid (VIP 630) and lipids (VIPs 399, 425, 979 and 981). Using the absorption spectra, the carotenoid was identified as peridinin which exhibited a maximum wavelength at 473-475 nm^35,36^. For the 4 other VIPs, spectrum and proposed mechanisms of fragmentation in the literature leads to the putative identification of 3 VIPs among which two digalactosyldiacyglycerols (DGDG 18:5/20:5 and DGDG 20:5/18:4)^37,38^ and the brassicasterol^39^. Based on mass and calculated formula, the last VIP might be a sterol.

**Table 1:** Accurate mass measurements, molecular formulas, and putative identification of the 5 determined VIPs.

Ionic form	Experimental *m/z*	Calculated molecular formula	Calculated *m/z*	Mass error [ppm]	MS/MS fragmentation	Putative identification
[M + H]^+^	399.3624	C_28_H_47_O	399.3621	0.6	281.3509^a^, 297.2574, 255.2104, 135.1167	Brassicasterol
[M + H]^+^	425.3413	C_29_H_45_O_2_	425.3414	−0.3	407.3306^a^, 191.1064, 173.0959	Sterol
M°^+^	630.3539	C_39_H_50_O_7_	630.3551	−1.9	603.7519, 553.3241, 520.0495, 487.2805	Peridinin
[M + Na^+^]	979.5394	C_53_H_80_O_15_Na	979.5389	0.5	705.3445^b^, 677.3134^c^	DGDG 18:5/20:5
[M + Na^+^]	981.5551	C_53_H_82_O_15_Na	981.5546	0.6	679.3299^b^, 705.3453^c^	DGDG 20:5/18:4

^a^[M + H - H_2_O]^+^, ^b^[M + Na - FA_1_]^+^, ^c^[M + Na - FA_2_]^+^, FA = fatty acid.

## Discussion

This is the first study demonstrating that biofouling formation is strongly influenced by surrounding benthic sessile species. In particular, we show that in its most severe form, the resulting effect of a species-specific assemblage is biofouling inhibition. Biofouling is a complex sequential process, which can be modulated by many abiotic and biotic factors, ranging from external factors to intrinsic influence of the marine taxa participating in biofouling formation itself.

Among these factors, nutrients are known to influence growth and productivity of various organisms such as bacteria and algae. In our study, nutrient contents in the seawater of each aquarium were compatible with those observed in previous nutrient analyses from the Moorea lagoon^26,40^. An increase of silicates was notably observed during the wet season as well as after strong rains^41^, as was the case for all aquariums of the A experiment. This link with external parameters can be explained by the design of our experiment, which consisted of an open-circuit with a water flow of 20 L/h per aquarium of 40 L. Therefore, the lagoon seawater composition mainly accounted for the significant variation of nutrient composition between the 3 experiments. As the effect of species assemblage on biofouling development was reproducible, this nutrient discrepancy between experiments could not account for the observed differences in biofouling development.

Studies have shown that bacteria, plankton and phytoplankton as well as dissolved organic and inorganic molecules are filtered from seawater and ingested by corals and bivalves^42,43^. This feeding can lead to a depletion of 30 to 45% of total chlorophyll a and picoplankton above a scleractinian coral dominated reef when compared with adjacent waters^42^. *Tridacna* species are able to filter up to 58% of algal cells from their surrounding water^44^. Moreover, a recent study has shown that corals are able to feed on diatoms such as *Thalassiosira* sp.^43^. In light of these results, and taking into account that giant clams filter large amounts of seawater (e.g. water filtration rate of one *Tridacna crocea* is 2-3 L/h)^44^, filtering activities could reduce the number of species participating in biofouling development and so could disrupt some steps of biofouling formation. Therefore, whenever another heterotrophic species is added to an assemblage, the community of microorganisms within the aquariums’ seawater could be modified (e.g. additive depletion of microorganisms). Such modifications could account for the differences in biofouling composition and development observed between assemblages, the stronger impact of these additive depletion effects leading to the inhibition of biofouling (e.g. lack of key species required for biofouling progression). Biofouling development was strongly inhibited in the three species assemblages, PAT, of which aquarium water was depleted of 47 bacteria, 2 diatoms and 3 algae genera when compared to the other assemblages. Among these lacking organisms, some bacterial taxa e.g. Rhodobacteraceae that are important biofilm precursors^45^, could be required for effective biofouling formation. Further studies of giant clam and coral filter-feeding species interactions will be helpful to better understanding their respective putative roles in depleting the microorganisms involved in biofouling progression. However, our experimental conditions with high seawater renewal and successive samplings led to four organisms per species at the end of the experiments. Therefore, corals and giant clams’ nutrition alone cannot be responsible for the disappearance of biofouling key species. Even though relevant, this hypothesis is not sufficient to account for the observed difference in biofouling development.

In contrast to ingested filtered organisms, those associated with invertebrate species, bacteria and microalgae, have been well characterized. Bacteria have been found living on the surface, in the tissues and in the mucus of coral species^46–48^. Because mucus contains a lot of inorganic phosphate and dissolved organic carbon, it is suspected as an effective inducer of bacterial growth^49^. Moreover, the diversity and quantity of the hosted bacteria differs not only between species but also according to their environment^50^. Dobrestov and collaborators^12^ have shown that numerous bacteria associated with corals possess an anti-bacterial activity, and antibacterial compounds in coral mucus have notably been found to select specific bacterial population leading to an antifouling effect^51^. Interestingly, 7 bacterial species were specifically found in PAT assemblages. Among them, bacteria belonging to the genus *Lysobacter* which are able to degrade biofilm and could contribute to the observed antifouling effect^52^. Corals and giant clams are known to produce bioactive compounds such as sterols which are described as chemical defensive substances^53,54^. Indeed, living corals are free of fouling and this particularity come from the production of secondary metabolites, especially sterols playing key roles in allelopathy with the ability to inhibit the growth of organisms around them^54^. Thereby, a fouling-resistant composition, non-toxic for the environment and applicable on immersed equipment, was made with sterols^55^. Even though no specific metabolite to the PAT assemblage was detected, suggesting that no new compound is produced in this context, 5 VIPs characterizing the PAT assemblage were highlighted. Among them, one was assigned to carotenoids and 4 to lipids. The carotenoid, identified as peridinin, is a specific pigment found in *Symbiodinium* dinoflagellates from corals and giant clams^35^. Interestingly, like most of the carotenoids, peridinin exhibits an inhibitory activity even at low concentration. As peridinin can inhibit cell proliferation and can exhibit a cytotoxic activity^56,57^, it might contribute to the biofouling inhibition observed in PAT assemblage. Moreover, among the lipids identified, one was assigned to brassicasterol and two to DGDG. Brassicasterol is found in various marine organisms such as cnidarians, sponges or diatoms^58–60^. In addition to having significant anti-inflammatory properties^60^, this sterol exhibits activity against organisms such as protozoans^61^. Furthermore, DGDG are also found in marine organisms, especially in *Symbiodinium* from both corals and giant clams^62,63^. Digalactosyldiacylglycerols possess strong nitric oxide inhibitory properties leading to antiviral activity^64,65^. Moreover, DGDG have been identified as antifouling agent demonstrating repellent activity notably of blue mussels^66^. These data suggest that a synergism of secondary metabolites might lead to antifouling activity as it has already been assumed in a previous study^67^. Therefore, in the present study, either due to their abundance as VIPs or to their synergism, carotenoid and lipids might be responsible for the biofouling inhibition specifically observed in PAT assemblage. In this regard, as mucus is continuously released by both corals and giant clams^68^, we cannot exclude that mucus flocs and/or secreted molecules such as antibiotics could adhere to the aquarium’s walls, playing a role in biofouling production. This effect will be, as discussed for nutrition, dependent on the different species present in the aquarium, with possible positive or negative interactions.

Regardless of the mechanisms involved in this inhibition, our experimental results suggest that biofouling develops differently, depending on the type and complexity of the sessile species assemblages and this may have important implications in coral reef ecology. Moreover, these phenotypes were maintained throughout experimental trials, even during the five days short thermal stress, suggesting that occasional increases in seawater temperature are not sufficient to induce differences of biofouling appearances. Global warming, ocean acidification, and local human impacts are considered as the main causes of coral reefs deterioration globally^69–71^. The combination of these factors usually prevent resilience of coral reefs, and favour the shift from coral-dominated to algal-dominated reef ecosystems^72–74^. Our discovery of an assemblage dependent antifouling activity is all the more important that biofouling inhibition occurs even in brief increased temperature conditions. Another major point of our results is that the loss of one species in a three species assemblage leads to a change in the algal inhibition spectrum, the two species assemblage being less active against or more prone to algal development. Despite the timeliness of understanding ecological shifts towards algal-dominated tropical habitats, a gap of knowledge still exists about the mechanisms governing these shifts^75^. It is well known that coral reefs taxa, e.g. scleractinian corals species, are differentially sensitive to environmental stressors^76–78^ and that, among sessile species, giant clams are notably more resistant than corals to sea water warming^79,80^. Therefore, environmental stress raises sequential and differential loss of species. This selective loss of species may impact the structuration of coral reefs benthic communities, leading to a change in the species interaction network and therefore, as exemplified by our work, might strongly impact their defence mechanisms against prerequisite steps of algal development. On the contrary, maintaining the diversity may temper this coral-algal shift. Hence, our study pinpoints that sessile coral reef species diversity, distribution and fitness might strongly influence this coral-algal shift, underlining the putative dramatic consequences of the decrease in biodiversity and the health of coral reefs.

Beyond the biofouling phenotypes, with its extreme form translating into biofouling inhibition, the present study also highlighted the functional interactions resulting from sessile species assemblages. Our data showed that specific communities of bacteria, diatoms and algae characterized the biofouling composition of each assemblage. The bacterial functional profiling revealed an increase of “stores polyhydroxybutyrat” in PA assemblage that could be linked to a need of energy storage molecule or to a stress^81^. In line with this interpretation, microbiomes in PA assemblages included species of *Pseudoalteromonaceae* that are able to degrade a form of polyhydroxybutyrat referred to as poly-3-hydroxybutyrate^82^. Other functions such as sulfur reducer, iron oxidizer, streptomycin producer and cellobiose degrading were, respectively, strictly found in T, PAT, P and PA. Additionally, some specific functions such as nitrite reducer and atrazine metabolism for T and PAT or degrade aromatic hydrocarbon for PAT, P and PA, are shared by some but not all of the assemblages. Therefore, the presence of nitrite reducer activities in both PAT and T assemblages could account for the systematically higher level of NH_4_^+^ in these assemblages. Such common features between some specific assemblages highlight the potential cumulative functional effect of the addition of one species in an assemblage, such as degrading aromatic hydrocarbons produced by P in PAT and PA as well as some emergent functions such as iron oxidizer in PAT. Therefore, all these combinations of functions provide a specific capacity to an assemblage for responding to changing environmental conditions. Such a trait can be related to the beneficial effect of plant assemblages for the growth, productivity and protection against pathogens and others, such as in permaculture where combinations of plants are used to enhance resistance and productivity^83^.

Considering the resulting functional aspects of various species assemblages, with some positive and/or negative interactions, together with their effect on biofouling development, our data support the notion that marine species assemblages should be integrally part of future coral reefs restoration plans as is already the case for terrestrial restoration.

## Supplementary information

Supplementary S1

Supplementary S2

Supplementary S3

Supplementary S4

Supplementary S5

Supplementary S6

Supplementary S7

Supplementary S8

Supplementary S9

## Data Availability

Supplemental information for this article can be found at http://www.nature.com/scientificreports. All data are available upon request.
