# Supplementary material for: Differential effects of coral-giant clam assemblages on biofouling formation
Source: Sci Rep. 2019 Feb 25;9:2675. doi: 10.1038/s41598-019-39268-1 (PMC6389951; doi:10.1038/s41598-019-39268-1)
Supplement: Supplementary file 1 — Supplementary S1 [file 41598_2019_39268_MOESM1_ESM.pdf]

Supplementary S1

Differential effects of coral-giant clam assemblages on biofouling formation

Isis Guibert, Isabelle Bonnard, Xavier Pochon, Mayalen Zubia, Christine Sidobre, Gaël Lecellier and Véronique Berteaux-Lecellier.

Table of contents

S1a: Experimental design……………………………………………………………………………………………2

S1b: Studied samples………………………………………………………………………………………………4

S1c: Detailed Materials and methods…………………………………………………………………………5

Biofouling observation and cytology……………………………………………………………………5
DNA extraction, PCR amplification and sequencing………………………………………………5
Metabolomic sample preparation and Liquid Chromatography-Mass Spectrometry (LC-MS) analysis.……………………………………………………………………………………………………5
Data analysis……………………………………………………………………………………………8
Cytology data analysis……………………………………………………………………………………8
Nutrients data analysis……………………………………………………………………………………8
Metabarcoding data processing pipeline……………………………………………………………8
LC-MS data pre-processing and metabolomic statistical analysis…………………………9
References……………………………………………………………………………………………………10

S1a: Experimental design

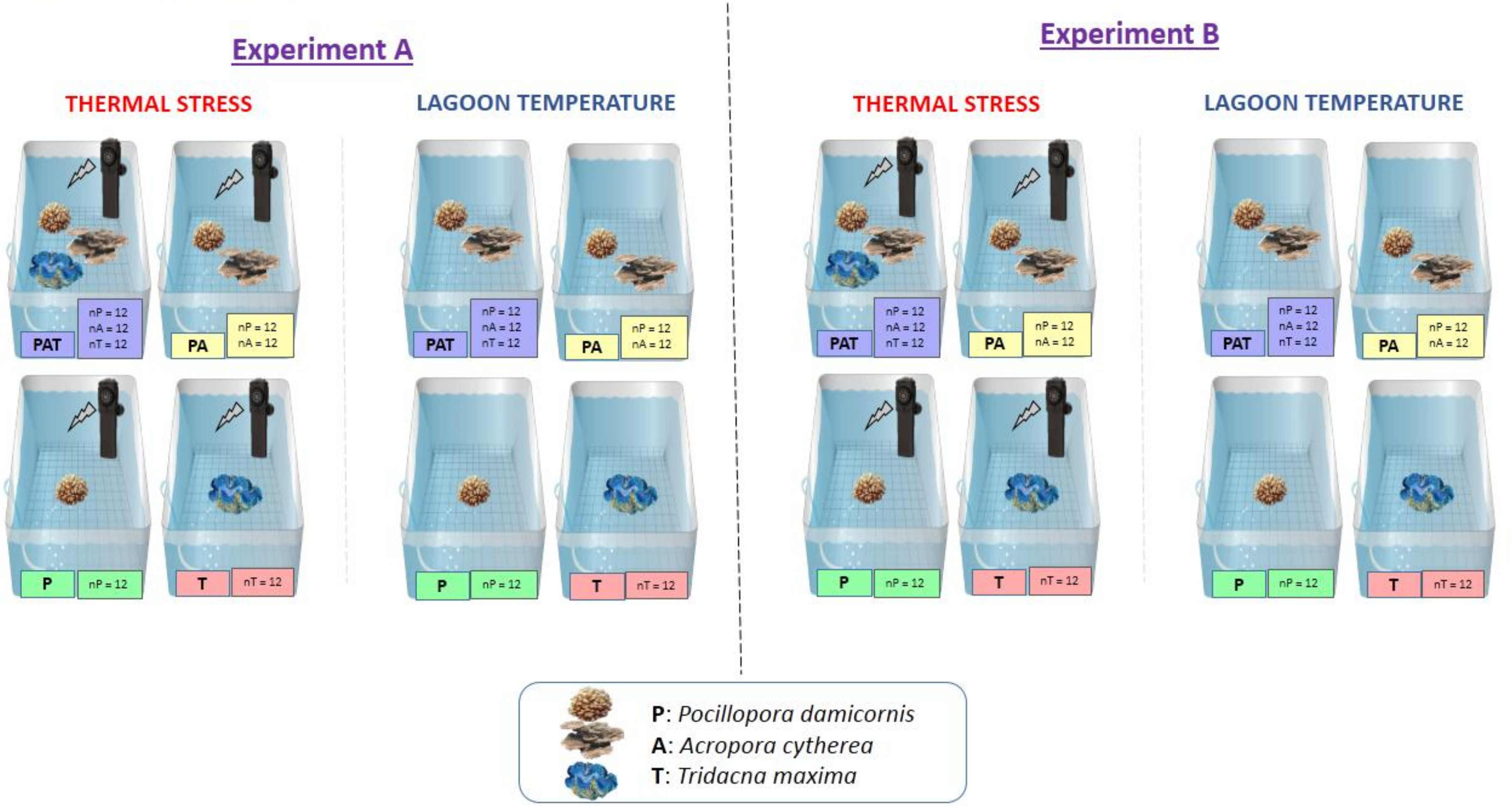

S1a: Experimental design

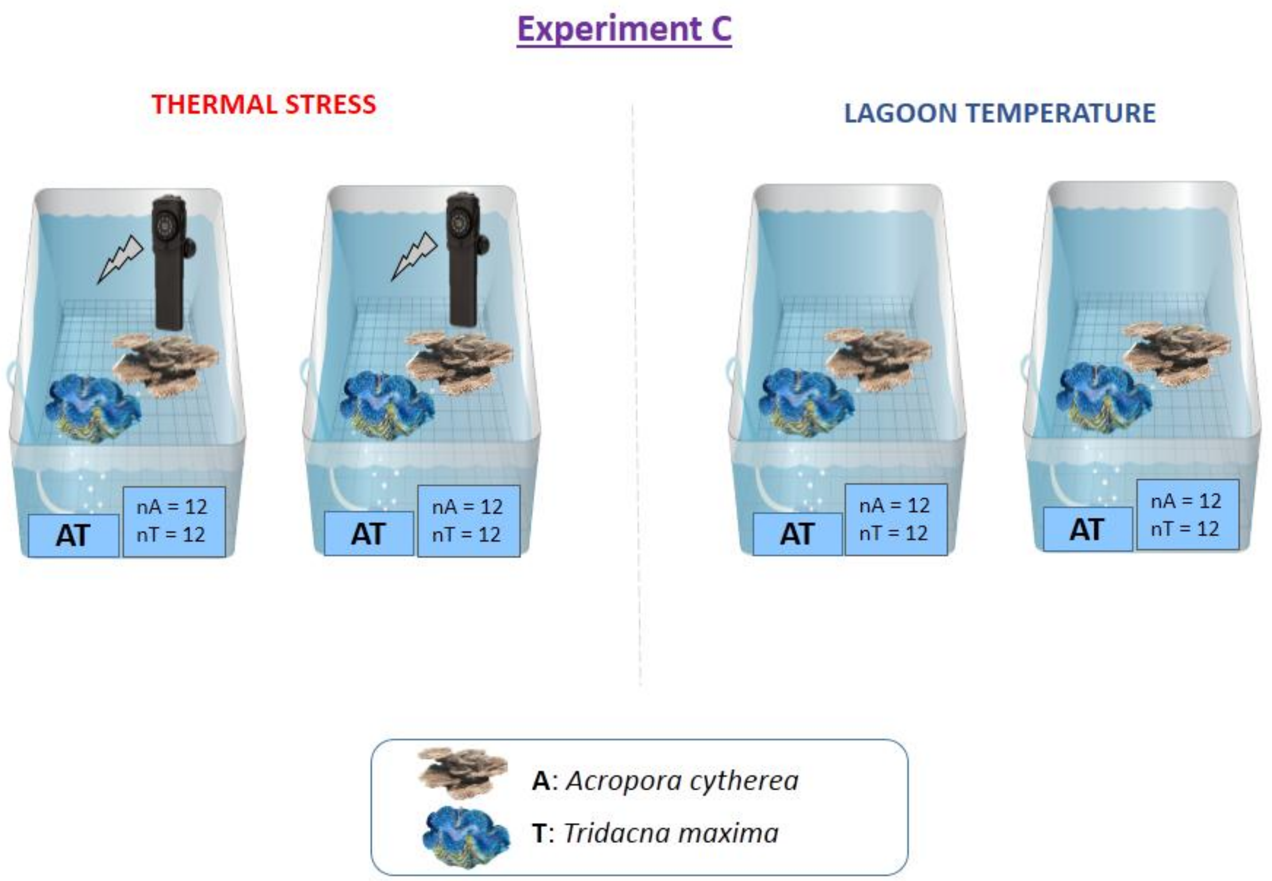

Supplementary 1a was realized by Guibert Isis with the use of pictures from Guibert Isis, and aquarium tank and airstone drawing from Tracey Saxby, Integration and Application Network, University of Maryland Center for Environmental Science (ian.umces.edu/imagelibrary/).

<u>**S1b: Studied samples**</u>

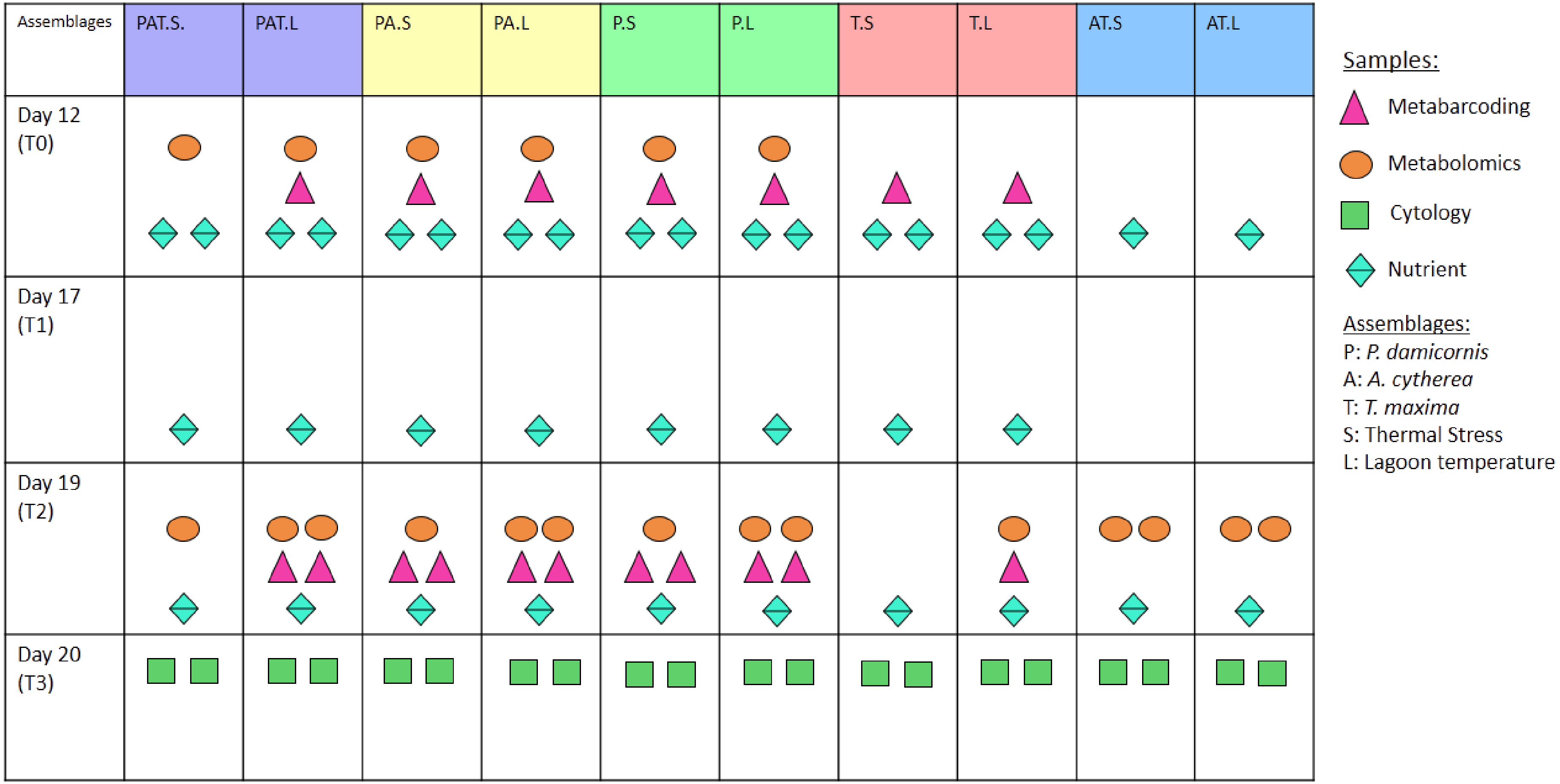

S1c: Detailed Materials and methods

Biofouling observation and cytology

Throughout the experiments, photographs of the aquarium were taken (days 15 or 19) using a Panasonic DMC-FT5 or a Go-Pro Hero 2 and daily observations of the biofouling were recorded.

At beginning of each experimental run, two sterile microscope cover glasses and two sterile glass slides were disposed in each aquarium. At the end of the experiments, microscope cover glasses were incubated in 7.4% paraformaldehyde for 15 minutes, rinsed twice with PBS X1 (80 mM Na_2HPO_4 and 20 mM NaH_2PO_4, 2 H_2O), and then disposed on a glass slide in one drop of mounting medium (Sigma, Kawasaki, Japan). Cover glasses were finally sealed on with nail polish. Concerning glass slides, a clean cover glass was directly disposed on their surface with a drop of mounting medium at the end of each experiment. All slides were observed using an Axio Imager microscope (Zeiss, Oberkochen, Germany) coupled with an EOS 100D camera, in order to identify the most predominant genera per assemblage.

DNA extraction, PCR amplification and sequencing

The polymerase chain reactions were performed in 50 µL volumes with the reaction mixture containing 5 µL GC enhancer, 1 µL of each primer (10 pmol/µL), 30 µl AmpliTaq Gold® 360 PCR Master Mix (Life Technologies, Carlsbad, California) and using 10 to 50 ng of template DNA. The PCR was performed under the follow thermocycling conditions: initial denaturation at 95 °C for 3 min, then 40 or 45 cycles consisting of denaturation at 94 °C for 30 s, annealing at 52 °C for 30 s, elongation at 72 °C for 90 s, and a final elongation at 72 °C for 7 min. PCR products were visualised on a 2% agarose gel.

Metabolomic sample preparation and Liquid Chromatography-Mass Spectrometry (LC-MS) analysis

Input pipe seawater (n=5) together with seawater from aquariums (n=23) were filtered using cartridges Strata-XL 100u polymeric reversed phase (Phenomenex, Torrance, California). Strata-XL cartridges were first conditioned with 40 mL ethanol–chloroform 1/1 (v/v) and then washed with 100

mL distilled water. Six litres of seawater per sample were filtered using an extraction chamber and the cartridges were washed again with 800 mL distilled water. They were left to dry for twenty minutes on the extraction chamber and finally stored at – 80 °C.

Samples were extracted from the Strata-XL cartridges with 10 mL dichloromethane-methanol 7/3 (v/v), then centrifuged 10 minutes at 3500 rpm to separate the residual water. The aqueous phase was discarded, and the organic phase was transferred in a glass hemolysis tube before drying under nitrogen flow and freeze-dried overnight. Samples were stored at – 20 °C.

LC-MS analyses were performed to determine metabolites in the seawater of each assemblages using an LCQ Fleet 2300 with an LC-ESI-MS system equipped with an electrospray ionization source, an Accela LC pump, and an ion trap from Thermo Scientific® (Waltham, Massachussetts). The organic extracts were separated using a Kinetex® C6-phenyl column (1.7 µ, 100 Å, 150 x 2.1 mm; Phenomenex) with a pre-column operating at 30 °C. The mobile phase was composed of water (A) and acetonitrile (B) (both with 0.1% v/v formic acid). A linear gradient was applied from 35% B held for 2 min to 65% B in 8 min, from 65% to 100% B in 20 min followed by 5 min of 100% B, then return to 35% B in 5 min and stay 5 min in post-run. The flow rate was 270 µL/min. The samples were analyzed using a positive ion mode with full scan MS window of 200-2000 *m/z*, recorded at a scan rate of 2 scans/s, the sample cone voltage was 4 kV, the capillary temperature 280 °C and the gas flows 29 and 5 arbitrary units (AU) for sheath and auxillary gas, respectively. A quality control strategy was carried out using a Latin square design for samples, quality control (QC) and methanol blank samples in order to reduce the potential error of instrumental drift. Five QCs and methanol blanks were analysed at the beginning of the sequence, every seven samples and at the end.

To determine the exact mass of secondary metabolites selected, LC-HRMS analyses were performed on a UHPLC system (Vanquish, Thermo Scientific) interfaced to a QTOF mass spectrometer (MaxisImpact I, Bruker Daltonics, Billerica, Massachussetts). The chromatographic separation was

conducted on the same column than for LC-MS analyses (Kinetex C6-phenyl column) thermostated at 30 ˚C. The mobile phases were (A) water and (B) acetonitrile, both with 0.1% v/v formic acid. The gradient elution program was: 0 min, 50% B; 2 min, 50% B; 15 min, 100% B; 20 min, 100% B; 22 min, 50% B; 30 min, 50%B. The flow rate was 0.5 mL/min and the injection volume 5 µL.

The QTOF MS system was equipped with an electrospray ionization source (ESI) and operated in positive mode over the range *m/z* 80−1600 with a scan rate of 0.8 Hz. Source parameters were: capillary voltage, 300 V; nebulizer pressure, 43.5 psi (N_2); drying gas, 120 psi (N_2); and drying temperature, 200 °C. A QTOF external calibration was daily performed with a sodium formate solution, and a segment (0.1−0.25 min) in every chromatogram was used for internal calibration, using a calibrant injection at the beginning of every run. The sodium formate calibration mixture consists of 10 mM sodium formate in a mixture of water/isopropanol (v/v, 1:1). The instrument provided a typical resolving power of 36000−40000 during calibration (39274 at *m/z* 226.1593, 36923 at *m/z* 430.9137, and 36274 at *m/z* 702.8636).

To strengthen the putative identification of selected metabolites, LC-HRMS and LC-HRMSMS analyses were carried out on an Thermo Scientific Dionex Ultimate 3000 UHPLC system (composed with a Dionex Ultimate 3000 diode array detector, a Vanquish pump and autosampler, Thermo Scientific, San Jose, CA, USA) with the same column, same elution sytem, and same operating conditions than for LC-MS analyses. MS analyses were conducted on a Q-exactive Plus Hybrid Quadrupole-Orbitrap mass spectrometer (Thermo Scientific) equipped with a HESI source. Source parameters were as follows : spray voltage positive polarity 3.5 kV, capillary temperature 320 °C, auxiliary gas temperature 200 °C, gas flows 35 and 10 AU for sheath and auxiliary gas respectively. Full MS scans were aquired over the range *m/z* 200−2000 with a mass resolution of 35,000 and a AGC target at 1.10^6. In MS2, mass resolution was 17,500, AGC target 2.10^5, maximum ion fill time 200 ms, isolation window 4.0 *m/z*, normalized collision energy 25 and 35. Parallel reaction monitoring was targeted on the VIP mass list. A lock mass (*m/z* 391.28429-$C_{24}H_{38}O_4$-diisooctylphthalate) has been used as internal standard for mass

accuracy enhancement during spectral data acquisition.

Data analysis

As the observed biofouling was similar independently of thermal conditions and replicates, all data issued from same assemblages were pooled for each data analysis.

All statistical analysis were performed with the R v3.4.0 or v3.2.5 (for metabolomics) statistical computing environment [1] with appropriate packages mentioned above.

Cytology data analysis

A presence/absence matrix of the listed taxa according to the assemblage was created (vegan package). In order to evaluate the community variations between assemblages, we used a classical multidimensional scaling (MDS, stat package) analysis based on a dissimilarity matrix constructed with the Jaccard index [2].

Nutrients data analysis

A Principal Components Analysis on log + 1-transformed environmental data as well as ANOVA with experiment and aquarium factors as random variables and the post hoc Tukey test were performed.

Metabarcoding data processing pipeline

To produce Operational Taxonomic Units (OTUs) and associated statistics, sequence data were analysed following the Miseq standard operating procedure (SOP) using MOTHUR (v1.39.5; http://www.mothur.org/) [3]. Briefly, sequence reads were assembled into contigs and amplicons' adaptors were removed. After trimming sequences to improve quality (Quality Control>=35), they were split into two different groups according to the genetic markers (bacterial 16S and eukaryotic 18S). Unique sequences were selected and counted for each group. Using K-mer search method, sequences were aligned to the Silva full-length sequences and taxonomy references (Silva-vr128) [4].

Insertions or deletions were removed using the "screen.seqs" command and filtered to remove the overhangs at both ends of the sequences. Unique sequences were selected again to eliminate the potential redundancy created during sequence trimming. A pre-cluster step was used to split sequences in pre-clusters with up to 2 differences between sequences, and the VSEARCH algorithm was employed to remove chimeras [5]. Pre-cluster sequences were assigned (classify.seqs) using the Silva database with a cutoff level of 80, and mitochondria and chloroplast assignations were removed with the remove.lineage command. Operational Taxonomic Units were then clustered under a 0.03 cutoff level. The phylotypes of our sequences were determined according to their taxonomic classification. In order to go deeper in the taxonomy of the cyanobacteria, we created a reference database issued from top hit of the chloroplast sequences assigned by Silva database blasted to Genbank (Supplementary Table S9). Then, we assigned all these OTUs with the newly created database.

To analyze the bacterial and algal communities in the assemblages, lists of OTUs assigned at different taxonomic levels were created and visualized on the Venny website (2.1.0, http://bioinfogp.cnb.csic.es/tools/venny//index.html) [6].

Using the files created with the phylotype analysis, the bacterial function and community assemblages were determined using METAGENassist [7]. After filtering our data on interquartile range [8], 465 variables out of 912 were normalized over sample by sum and over taxa by range scaling. Oxygen requirement and metabolism of bacteria were visualized with a heatmap using Euclidean distance measure and the Ward's clustering method [9]. OTUs and reads Tables used for the analysis are available in the Supplementary Tables S4-S6.

LC-MS data pre-processing and metabolomic statistical analysis

LC-MS raw data were converted to netCDF files with Xconvert from Thermo Scientific™ Xcalibur™ software (version 2.1). The data were analysed with an XCMS pipeline using R (XCMS version 1.46.0, R

version 3.2.5) [10]. Several steps were necessary for XCMS processing with the following parameters: feature detection (step = 0.2, mzdiff = 0.4, fwhm = 30, snthresh = 30), time range reduction (c(360, 1920)), alignment (minfrac = 0.20, mzwid = 0.25, bw = 30 and 10), retention time correction (profstep = 1, method = obiwarp). The data matrix of the variables corresponding to metabolites was made using a diffreport with the XCMS package. Before statistical analysis, quality of the samples was investigated using the quality controls and methanol blanks.

A Partial Least Square Discriminant Analysis (PLS-DA, MixOmics package) [11] was performed to identify the molecules that best distinguished the different assemblages. Quality controls and methanol blanks were removed for the statistical analysis. Metabolites which separate PAT assemblage from the other were selected using a cosx>0.2 & cosy>0.2, a cosx>0.5 or a cosx>0.5 & cosy>0.001. From these metabolites, VIPs (Variable Importance in the Projection) abundant in PAT assemblage were selected from individuals' boxplot.

References

1. R core team. A language and environment for statistical computing. (2014).

2. Jaccard, P. The distribution of the flora in the alphine zone. *New Phytol.* **XI,** 37–50 (1912).

3. Kozich, J. J., Westcott, S. L., Baxter, N. T., Highlander, S. K. & Schloss, P. D. Development of a dual-index sequencing strategy and curation pipeline for analyzing amplicon sequence data on the miseq illumina sequencing platform. *Appl. Environ. Microbiol.* **79,** 5112–5120 (2013).

4. Pruesse, E. *et al.* SILVA: A comprehensive online resource for quality checked and aligned ribosomal RNA sequence data compatible with ARB. *Nucleic Acids Res.* **35,** 7188–7196 (2007).

5. Rognes, T., Flouri, T., Nichols, B., Quince, C. & Mahé, F. VSEARCH: a versatile open source tool for metagenomics. *PeerJ* **4,** 2584 (2016).

6. Oliveros, J. . Venny. An interactive tool for comparing lists with Venn's diagrams. (2015).

7. Arndt, D. *et al.* METAGENassist: A comprehensive web server for comparative metagenomics. *Nucleic Acids Res.* **40,** 88–95 (2012).

8. Hackstadt, A. J. & Hess, A. M. Filtering for increased power for microarray data analysis. *BMC Bioinformatics* **10,** 11 (2009).

9. Ward, J. H. Hierarchical Grouping to Optimize an Objective Function. *J. Am. Stat. Assoc.* **58,** 236–244 (1963).

10. Smith, C. a *et al.* XCMS: processing mass spectrometry data for metabolite profiling using Nonlinear Peak Alignment,Matching,and Identification. *Anal. Chem.* **78,** 779–87 (2006).

11. Rohart, F., Gautier, B., Singh, A. & Lê Cao, K.-A. mixOmics: An R package for ‘omics feature selection and multiple data integration. *PLOS Comput. Biol.* **13,** e1005752 (2017).
