# Supplementary material for: Differential effects of coral-giant clam assemblages on biofouling formation
Source: Sci Rep. 2019 Feb 25;9:2675. doi: 10.1038/s41598-019-39268-1 (PMC6389951; doi:10.1038/s41598-019-39268-1)
Supplement: Supplementary file 3 — Supplementary S3 [file 41598_2019_39268_MOESM3_ESM.pdf]

Supplementary Figure S3

Isis Guibert, Isabelle Bonnard, Xavier Pochon, Mayalen Zubia, Christine Sidobre, Gaël Lecellier and Véronique Berteaux-Lecellier.

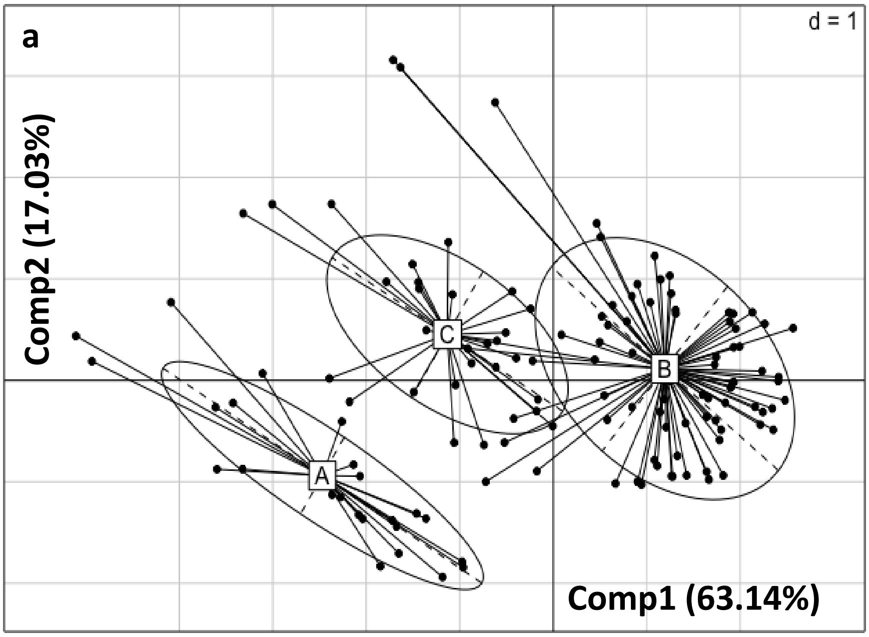

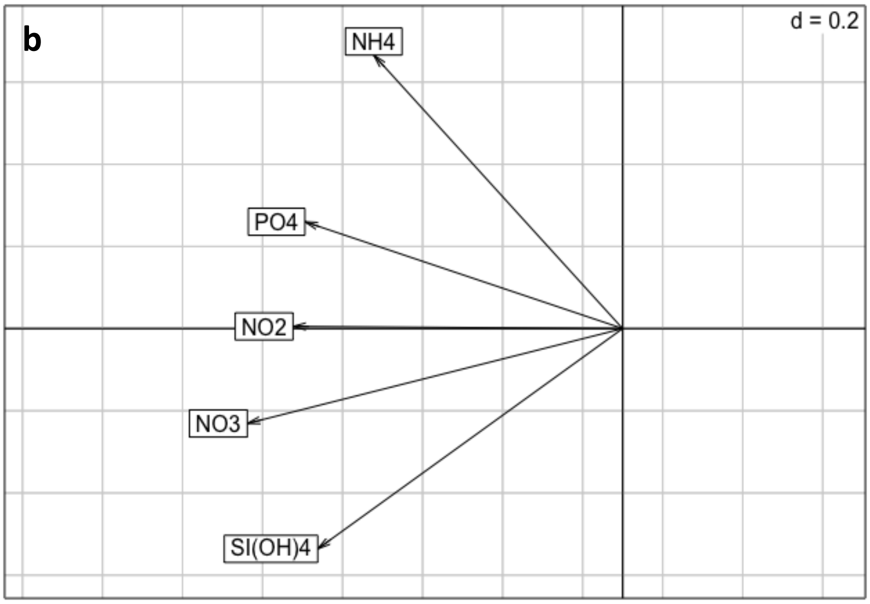

Figure S3

Principal components analysis of seawater nutrients. **(a)** Individual factor map, A,B and C: name of the 3 experiments **(b)** Variables factor map; ammonium (NH4), phosphate (PO4), nitrate (NO2), nitrite (NO3), silicate (SI(OH)4).
