# Supplementary material for: Differential effects of coral-giant clam assemblages on biofouling formation
Source: Sci Rep. 2019 Feb 25;9:2675. doi: 10.1038/s41598-019-39268-1 (PMC6389951; doi:10.1038/s41598-019-39268-1)
Supplement: Supplementary file 7 — Supplementary S7 [file 41598_2019_39268_MOESM7_ESM.pdf]

Supplementary Figure S7

Isis Guibert, Isabelle Bonnard, Xavier Pochon, Mayalen Zubia, Christine Sidobre, Gaël Lecellier and Véronique Berteaux-Lecellier

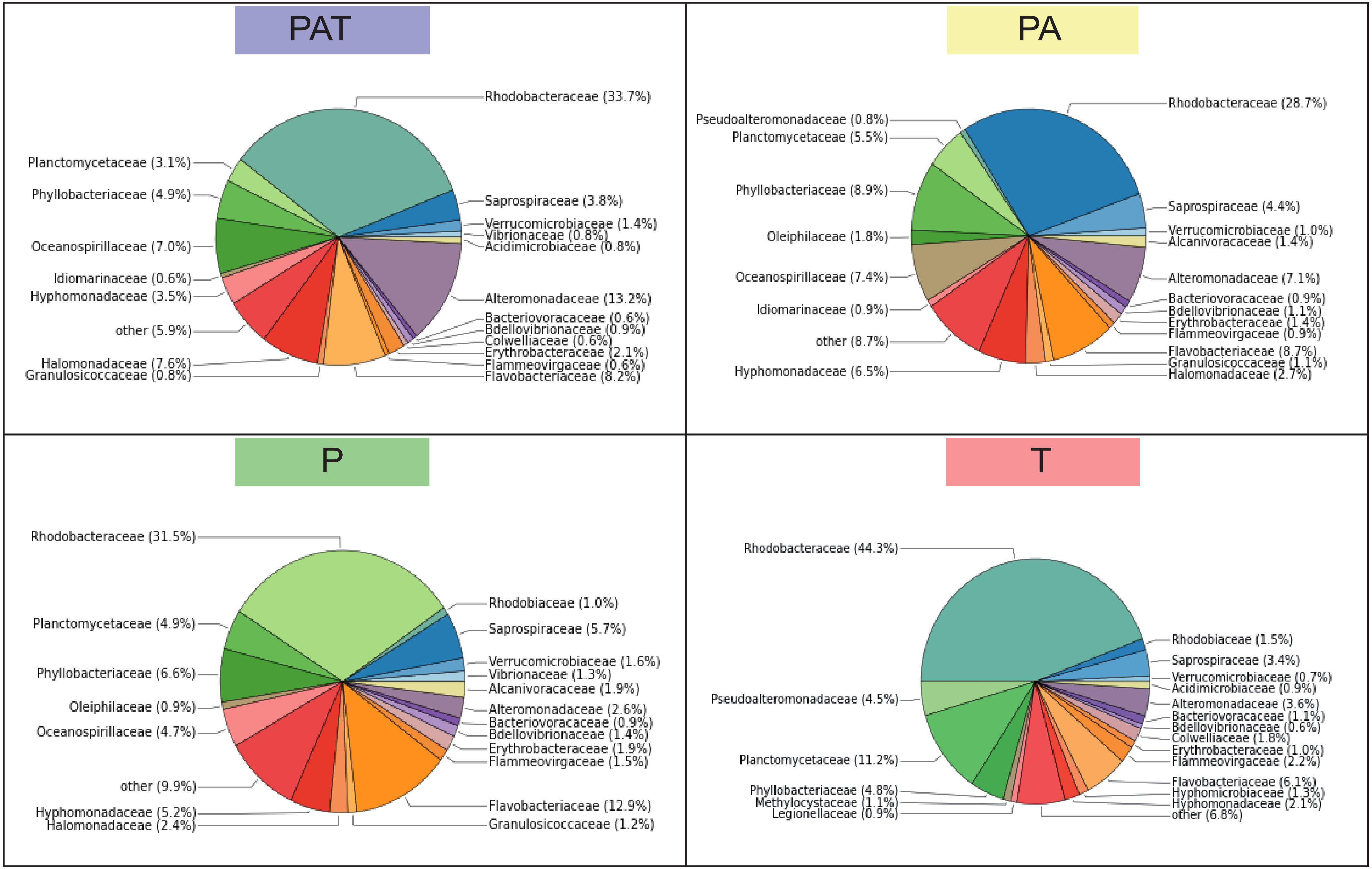

Figure S7

Pie Chart of bacteria families in each assemblage. PAT: P. damicornis, A. cytherea, T. maxima; PA: P. damicornis, A. cytherea; P: P. damicornis; T: T. maxima.
