# Supplementary material for: Differential effects of coral-giant clam assemblages on biofouling formation
Source: Sci Rep. 2019 Feb 25;9:2675. doi: 10.1038/s41598-019-39268-1 (PMC6389951; doi:10.1038/s41598-019-39268-1)
Supplement: Supplementary file 8 — Supplementary S8 [file 41598_2019_39268_MOESM8_ESM.pdf]

Supplementary Figure S8

Isis Guibert, Isabelle Bonnard, Xavier Pochon, Mayalen Zubia, Christine Sidobre, Gaël Lecellier and Véronique Berteaux-Lecellier.

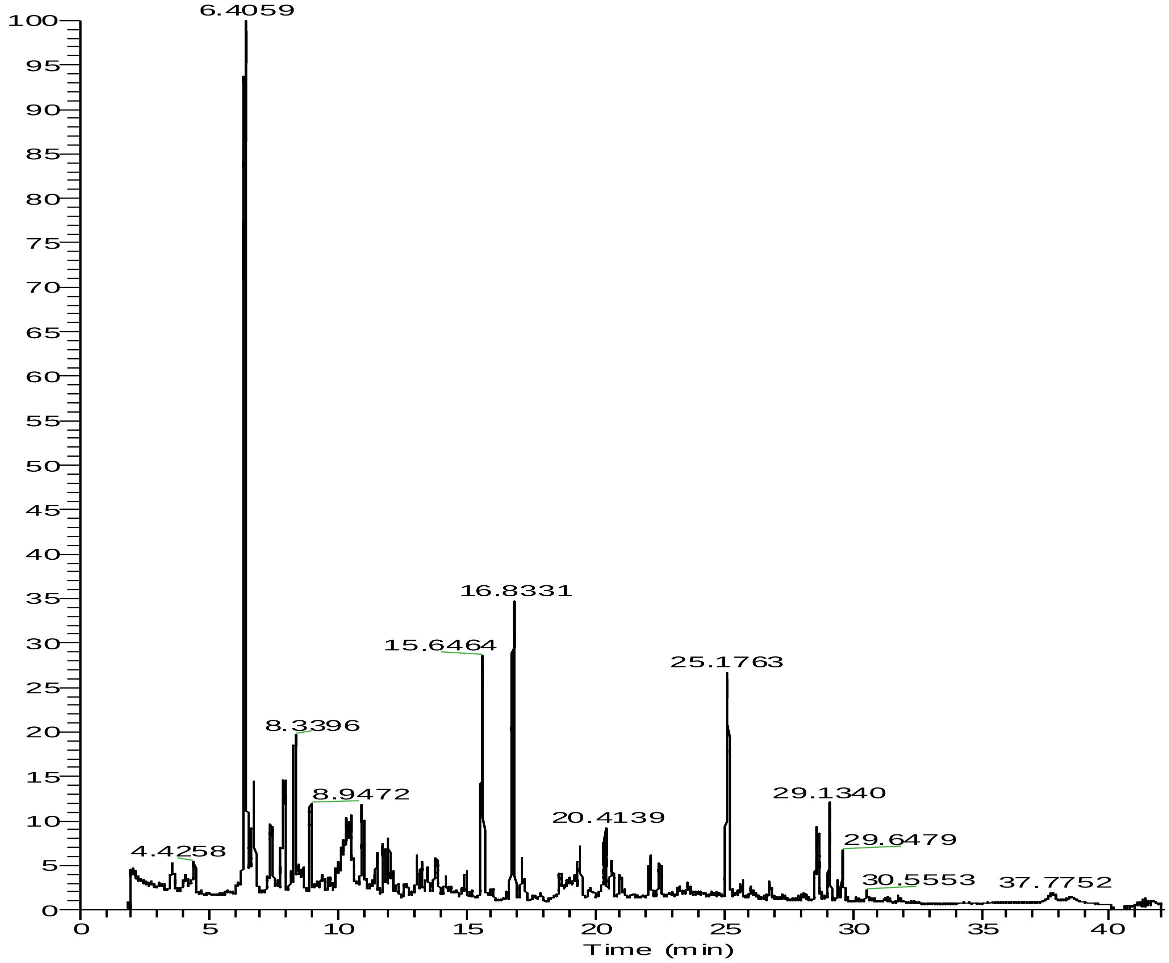

Figure S8

Representative LC-MS profile of seawater from PAT assemblages. Total ion chromatogram (TIC) from ESI+ analysis on a LCQ Fleet sytem. PAT: *P. damicornis*, *A. cytherea* and *T. maxima*.
